# From the dual-dimensional perspective of employee mindfulness and superior trust, explore the influence mechanism of negative workplace gossip on work engagement

**DOI:** 10.3389/fpsyt.2023.1287217

**Published:** 2023-11-24

**Authors:** Xiaoli Cheng, Jiaxin Duan, Weilin Wu, Lei Lu

**Affiliations:** ^1^Jinhua Open University, Jinhua, China; ^2^School of Business, Macau University of Science and Technology, Taipa, Macau SAR, China; ^3^School of Economics, Institute for China Common Prosperity Research, Jinxing University, Jinxing, China; ^4^School of Psychological and Cognitive Sciences, Beijing Key Laboratory of Behavior and Mental Health, Peking University, Beijing, China

**Keywords:** mindfulness, negative workplace gossip, professional commitment, superior trust, work engagement

## Abstract

**Introduction:**

As a common phenomenon of workplace negative gossip in organizations, how it affects employees’ work engagement is not yet clear, nor what methods can be used to mitigate its negative impact on employees’ work engagement.

**Methods:**

Based on Conservation of Resource Theory, this study obtained 334 valid employee samples from mainland China enterprises through a three-time lagged research design and explored the mechanism of negative workplace gossip on work engagement from the dual perspectives of employees and supervisors.

**Results:**

The results show that: (1) Negative workplace gossip negatively affects employee work engagement. (2) Professional commitment plays a mediating role between negative workplace gossip and employee work engagement. (3) Employee mindfulness negatively moderates the negative impact of workplace negative gossip on professional commitment; superior trust negatively moderates the negative impact of workplace negative gossip on professional commitment. (4) Employee mindfulness and superior trust are further weakened to moderate the negative indirect impact of workplace negative gossip on employee work engagement through professional commitment, and this negative indirect impact is weaker when employees have a higher degree of mindfulness and higher trust in superiors.

**Discussion:**

It proposes effective strategies for managing workplace gossip to harness its positive influence and offer practical guidance to enhance employee work engagement.

## 1 Introduction

In the context of economic globalization, a complex and volatile market environment, and the rapid advancement of science and technology, an increasing number of organizations recognize that their employees are the source of their fundamental competitiveness (1). Whether employees can actively and proactively engage in work is crucial to the development of the organization. Employees are more energetic and fully committed to work, dare to accept work challenges and not give up easily, and achieve better performance for the organization and individuals (2). Work engagement is the key link connecting work factors and work performance, and it is a crucial strategy used by businesses to obtain a competitive edge (3). Therefore, enhancing workers’ work engagement is crucial for both the organization’s and the workers’ personal growth (4).

At the same time, with the intensification of workplace competition, the problem of workplace violence has also intensified, and scholars and managers have become more and more interested in the “dark side” of organizational behavior (5, 6). In the context of Chinese organizations, influenced by traditional culture and implicit personal characteristics, workplace violence mostly occurs in the form of workplace gossip (7). Gossip is ubiquitous. In daily life, people often hear, participate in or spread other people’s gossip intentionally or unintentionally, and use it as an important way to obtain information, vent emotions and maintain relationships (8). In the specific social situation of the workplace, positive and negative gossip can be distinguished in the workplace, with the latter’s prevalence and worry rates being of more concern (9–11). Negative workplace gossip is an informal communication phenomenon that negatively evaluates an absent member (12). At present, most of the research studies and measures negative gossip in the workplace from the perspective of gossip goal perception, and explores the results of negative gossip in the workplace (13). Research has indicated that unfavorable rumors in the workplace have an adverse effect on workers’ behavior at work (14), and adversely impacts the organization’s performance. On the one hand, it will cause trouble to employees, and on the other hand, it will not be conducive to creating a good working atmosphere, which will adversely affect the performance of employees and the organization.

With the improvement of material living standards and the background of organizations advocating “people-oriented” humanized management, it is imperative to allocate increased focus towards the psychological well-being of employees (15). Work engagement is a positive, proactive, and energetic work state (16). The state of positive work engagement has a significant impact on employees’ proactive behavior and work performance. It enables them to effectively navigate the complex and dynamic working environment, thereby maintaining their competitiveness (2, 17). This study follows the trend of paying attention to employees’ mental health, combines negative gossip in the workplace with employees’ work engagement, and explores how negative workplace gossip affects employees’ work engagement in the context of common and frequent adverse interpersonal relationship situations and negative events within the organization. Simultaneously, this study takes the gossiped employees as the object and explores whether there are intervention methods to alleviate the impact mechanism of negative workplace gossip on employees’ work engagement. From the dual perspectives of employees’ own mindfulness and superior trust, this paper tries to clarify the function and mechanism of negative gossip in the workplace affecting professional commitment, which again affects employees’ work engagement.

In the context of workplace stressors, negative gossip can be fundamentally perceived as a potential threat to resource depletion (18). Grounded in the Conservation of Resources Theory, individuals inherently strive to acquire, preserve, and safeguard their personal resources. In cases where one’s resources are diminished and not easily replenished, individuals may resort to reducing resources to safeguard what remains (19). Consequently, as per the Conservation of Resources Theory, negative workplace gossip can lead to the depletion of an employee’s personal resources, thereby impacting their commitment and engagement in their work (20). At the same time, negative workplace gossip will destroy the emotional bond between employees and the organization, affect employees’ judgment of the organization, reduce professional commitment, and thus reduce their commitment to work (21). Although negative gossip in the workplace contributes to employees’ negative emotions and experiences, its effect is also influenced by several factors (22). Most of the existing studies on the volatilization effect of negative gossip in the workplace focus on the single-factor adjustment at the team or individual level (10, 11), which fails to comprehensively consider the synergistic influence of external and internal resources from the perspective of the integration of employees and superiors. Therefore, this study will introduce superior trust as an external resource and employee mindfulness as an internal resource, and comprehensively investigate the influence of the moderating effect (22). In order to make up for the shortcomings of existing studies, from the perspective of employees and leaders, this paper explores the moderating effects of superiors’ trust and employees’ mindfulness on workplace negative gossip and work engagement.

In summary, this study examines the impact and mechanisms of negative workplace gossip on employees’ work engagement, drawing on the Conservation of Resources Theory and considering the perspectives of both employees and their superiors. Initially, the study seeks to ascertain the impact of negative workplace gossip on employees’ level of work engagement, thereby contributing to a more comprehensive understanding of the consequences associated with negative workplace gossip. Furthermore, in conjunction with the conservation of resources theory, professional commitment serves as the mediating variable in examining the mediating role between negative gossip and work engagement. In this study, we aim to examine the moderating role of employee mindfulness and superior trust from the perspective of employees and leaders. Specifically, we will investigate how these two variables influence the relationship between negative workplace gossip and career commitment, focusing on their inhibitory effect. This study presents a moderated mediation model that aims to elucidate the impact mechanism of workplace negative gossip on employee work engagement within Chinese organizations. The model integrates both mediation and moderation effects, with the objective of providing guidance and inspiration for management practices in this context.

## 2 Hypothesis development

### 2.1 The relationship between negative workplace gossip and work engagement

Work engagement is a constructive and gratifying condition at work distinguished by three fundamental attributes: energy, dedication and focus (2). Work engagement means that individuals can maintain a high degree of physiological involvement in work and maintain a high degree of cognitive arousal. The necessary prerequisite for this is sufficient emotional and psychological resources (23). According to the conservation of resources theory, individuals exhibit a proclivity to uphold, safeguard, and obtain resources (24), and the loss of resources will lead to negative behaviors of employees (19). Specifically, negative gossip in the workplace makes employees feel isolated, worsens the interpersonal relationship among employees (8, 25), and increases the uncertainty and instability of work tasks (26), leading to serious depletion of employees’ emotional and psychological resources (27). The depletion of emotional and psychological resources has a detrimental impact on employee enthusiasm and dedication, posing challenges for employees in fully engaging with their work. In addition, negative gossip makes relevant employees unable to feel the importance and support of the organization and other members, thus losing enough energy to devote themselves to work (28). Secondly, the conservation of resources theory is that when individual resources are depleted, it will trigger a series of subsequent resource protection responses (19). Negative gossip in the workplace damages the personal reputation and image of employees, causing anxiety and negative emotions, consuming employees’ psychological resources, and making it difficult for employees to allocate more resources to work (29). At the same time, employees who are troubled by negative gossip in the workplace not only need to spend extra resources to trace and clarify the gossip information, but also be careful to avoid the spread of a new round of gossip, try to avoid dealing with interpersonal relationships and teamwork, and it is difficult to devote themselves to work (30). Finally, the presence of negative gossip within a professional setting has the potential to foster a sense of mutual distrust among employees, consequently leading to a negative emotional encounter for said employees (7), gossip can also easily lead to the loss of employees’ reputation, and cause the negative emotions of the gossip to expand (13), it is difficult to maintain a positive mental state, and it is difficult to engage in work satisfactorily. Therefore, this study proposes Hypothesis 1:

*H1*: Negative workplace gossip has a negative impact on employees’ work engagement.

### 2.2 Professional commitment as a mediator

Professional commitment reflects the degree of employee identification, commitment, and emotional attachment to the organization (31). Generally speaking, employees with high professional commitment are more identified with organizational goals, will take the initiative to make their own contributions to the organization, and actively devote themselves to work (32). Secondly, according to the social identity theory (33), the increase in employees’ identification with the organization will intensify the employees’ sense of belonging to the organization, their sense of identity and responsibility will be significantly enhanced, and eventually they will show more positive states and behaviors (34). Employees with high professional commitment are motivated by positive emotions, willing to fulfil organizational role expectations and put in extra work effort (35), and inject full personal cognitive, emotional, and physical commitment into their work (17). Thirdly, when the employee’s professional commitment reaches a certain intensity, the employee may actively engage in work and take active work behaviors in order to express their sincerity to the organization. Finally, Macey and Schneider (36) believed that the concept of work engagement contains emotional connotations, and proposed that professional commitment is an effective predictor of work engagement in achieving organizational goals (36).

Negative workplace gossip causes interpersonal stress, which drains emotional and psychological resources and lowers employees’ identification and investment in the company (37). According to the conservation of resources theory, when resources are reduced or threatened, individuals will become tense and exhausted, triggering uneasy interpersonal interactions and hindering the formation of professional commitment to the organization (27). First of all, employees who are subjected to negative gossip within the workplace are required to invest significant amounts of time and energy in order to effectively process and assimilate the adverse consequences resulting from such gossip (19). Due to the lack of psychological and emotional resources, employees cannot generate a professional commitment to the organization. Secondly, when employees perceive being attacked by gossip, they will greatly reduce their sense of obligation and responsibility to the organization (28), consuming their own professional commitment to the organization. Thirdly, employees suffer from negative gossip in informal communication, the need for emotional communication cannot be met, it is difficult to maintain emotional communication between employees and the organization, and it is difficult to form professional commitment (38). Finally, interpersonal emotional connection and psychological identity are clearly reflected in the organization. Negative gossip in the workplace causes emotional connection to be unsmooth (39). Emotional alienation intensifies (9–11), resulting in employees having difficulty attaching to and belonging to the organization’s professional commitment.

Therefore, based on the conservation of resources theory, this study believes that negative gossip in the workplace can make individuals fall into an unfriendly, unsafe, defensive and suspicious working climate, and employees are difficult to identify with and rely on the organization, and their career commitment is greatly reduced, resulting in employees’ inability to engage in work (14). At the same time, victims of gossip are affected by external negative influences such as personal image, reputation or career, and consume a large amount of their own resources, resulting in a sense of disappointment towards the organization, a decrease in the organization’s sense of identity and commitment, and an inability to work (40). Finally, the negative attributes of negative gossip in the workplace tend to lower the value standard and cause uneasy interpersonal interaction, resulting in a decline in employees’ attachment to the organization (41), and prompting employees to make negative perceptions and evaluations of the organization (42). These negative evaluations and experiences hinder the formation of employees’ identity and fulfilment of career commitments, consume their own emotional and psychological resources, and have a detrimental impact on employees’ work engagement (43). Therefore, this study proposes Hypothesis 2:

*H2*: Professional commitment mediates negative workplace gossip and work engagement.

### 2.3 Mindfulness as a moderator

Mindfulness is the quality of being conscious, non-judgemental, and focused on the present moment with openness and acceptance (44). As a positive personal trait, mindfulness not only has a positive and direct impact on the individual’s cognitive function, emotion regulation, and adaptive behavior, but also buffers the adverse effects of external risks (45). The Mindful Coping Model also believes that when individuals with high mindfulness face stressful events, they will make positive cognitive evaluations and re-evaluate stressful events, thereby reducing the negative impact of stress (46). Negative gossip in the workplace is regarded as a stressful situation, resulting in the loss of employees’ psychological and emotional resources, disapproval of the organization and team, and reduced professional commitment. In this negative gossip organization, employees use their own positive mindfulness traits to deal with the negative impact of stressful events; by adjusting their attitudes, they actively re-evaluate and define gossip events, reduce the impact of negative gossip, and maintain positive professional commitments (47).

First, individuals with high levels of mindfulness tend to interpret internal thoughts simply as mental events, weakening the need for external social approval (48). Furthermore, employees with high levels of mindfulness are less affected by external situations and are more inclined to consider themselves to completing work tasks (49). Therefore, employees with high mindfulness are less affected by negative gossip, which will also alleviate the impact of negative gossip on employees’ career commitment. Second, mindfulness can encourage individuals to pay attention to uncertainty and negative experiences with an open and accepting attitude (50). Correspondingly, the higher the level of mindfulness of employees, the higher the tolerance to uncertainty and the lower the occurrence of emotional problems (51). Employees with high mindfulness judge negative gossip events in the workplace with an open and accepting attitude, and alleviate the negative emotions and behaviors caused by negative gossip. Finally, employees with high levels of mindfulness have higher emotional regulation and self-acceptance, and can increase their psychological capital to cope with stress (52). When encountering negative gossip and stressful events in the workplace, employees with high levels of mindfulness are more likely to improve their mental health, regulate their emotions and tolerance, and maintain a positive emotional commitment to the organization. Therefore, this study proposes Hypothesis 3:

*H3*: Mindfulness negatively moderates the negative relationship between negative workplace gossip and professional commitment. The higher the level of employee mindfulness, the weaker the negative relationship between negative workplace gossip and professional commitment; otherwise, the stronger the relationship.

### 2.4 Superior trust as a moderator

The conservation of resources theory points out that the impact of resource acquisition and resource loss on individuals will show different effects due to the difference in individual initial resources (53). Negative gossip in the workplace, as a kind of interpersonal pressure, causes the loss of employees’ psychological and emotional resources and affects employees’ professional commitment. At the same time, in the context of Chinese organizations, superiors play a very important role. Since the structure of Chinese enterprises is not flat enough, the relationship between most employees and organizations is defined by the specific relationship with their superiors (54, 55). Therefore, it is necessary to clarify the impact of superiors as an external resource on the relationship between negative workplace gossip and professional commitment, and it is more in line with the actual situation of the organization.

Perceived superior trust refers to employees’ perception of superiors’ positive expectations of themselves and their willingness to take risks (56). In real work, superior leaders tend to have a lot of resources, and individuals’ perceived trust in superiors can not only obtain additional psychological resources, but also be supplemented by other work resources (57). Meanwhile, Employees who perceive trust from their superiors can supplement individual energy with relational energy (58). Therefore, the perceived trust of superiors can effectively measure the difference in initial resources, and may play a moderating role in the impact of negative gossip in the workplace on occupational commitment. Employees with higher levels of perceived trust are more psychologically resilient (59). This implies that individuals possess greater psychological capacities, enabling them to effectively manage adverse workplace rumors with composure, thereby minimising the detrimental impact of such rumors on employees’ dedication to their professional roles. On the contrary, employees with a low level of perceived trust from their superiors may pay more attention to negative workplace gossip related to themselves, pay more attention to the loss of resources caused by negative gossip, and undermine their own organizational identity and professional commitment (9–11). Furthermore, employees with a lower perceived level of trust from their superiors may devote more time and energy to understanding the sources of negative gossip (27), which leads to further resource damage, and thus the negative impact of negative gossip in the workplace on professional commitment becomes more intense. Therefore, hypothesis 4 is proposed:

*H4*: Perceived superior trust negatively moderates the negative relationship between workplace negative gossip and professional commitment. When employees perceive higher superior trust, the weaker the negative relationship between negative workplace gossip and professional commitment; otherwise, the stronger the relationship.

### 2.5 Mediation model with moderation

This study presents a moderated mediation model by incorporating the mediating effect of Hypothesis 2 and the moderating effects of Hypothesis 3 and Hypothesis 4. Employees are exposed to negative gossip in the workplace, which affects their professional commitment and makes it difficult to engage at work. Specifically, employees’ professional commitment plays a mediating role between workplace negative gossip and work engagement, but the mediating effect is influenced by employees’ own mindfulness and superiors’ trust. When employees have a higher level of mindfulness and perceive higher trust from superiors, the negative effects of workplace gossip on employees’ professional commitment and job engagement can be alleviated. Therefore, hypothesis 5 is proposed:

*H5*: Both employee mindfulness and perceived superior trust negatively moderate the indirect effect of workplace negative gossip on work engagement through professional commitment. When employees have a higher level of mindfulness and perceived higher superior trust, this indirect effect is weaker; on the contrary, this indirect effect is stronger.

**Figure 1:**
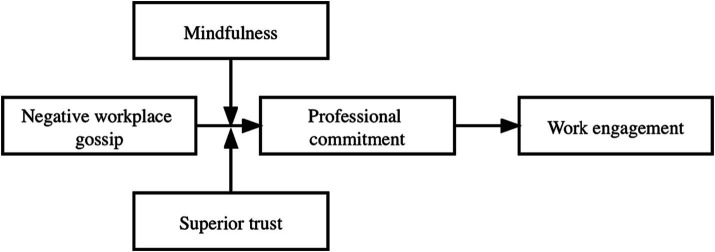
Proposed research model.

Based on the above theoretical hypotheses, this proposed research model is shown in Figure 1.

## 3 Research methods

### 3.1 Research subjects and collection procedures

In this study, a questionnaire survey was used to obtain data, and all questionnaires are pencil self-supporting reports. The data samples come from Beijing, Shanghai, Jiangsu, Zhejiang and other places, involving sales, marketing, finance, management and information technology departments. In order to prevent the influence of common method bias, the research time of longitudinal tracking was adopted in this study, and questionnaires were distributed in three time periods, with a time break of 1 month. Before commencing the formal investigation, our research team established an investigation group consisting of both MBA and DBA students, many of whom possess extensive executive experience. Additionally, we communicated with and provided clarification to the employees and supervisors who were participating in our study. We emphasized to all participants that there were no right or wrong answers and assured them of the anonymity and confidentiality of the questionnaires. Simultaneously, the participants were duly notified prior to the commencement of the formal inquiry that their involvement was exclusively intended for the purposes of this scholarly investigation. Finally, this project that collected the data from questionnaires was approved by the Institutional Review Board of the Peking University. An Ethic Issue Form offered by Peking University was signed and submitted to promise this article’s authenticity and compliance with academic ethics.

A total of 400 employees were invited to complete this study. In the first stage, employees fill out Questionnaire 1 to investigate negative workplace gossip; In the second stage, employees fill out Questionnaire 2 to investigate mindfulness, perceived superior trust, and professional commitment; in the third stage, employees’ work engagement is investigated. After the survey and research were completed, the last 4 digits of the mobile phone numbers of the employees were used as the matching basis for the three questionnaires, and invalid questionnaires that were omitted or wrongly filled were eliminated. Finally, this research obtained 334 valid questionnaires from mainland Chinese employees. The effective recovery rate was 83.5%. All samples are filled in by employees. The specific departments to which employees belong are as follows: 220 people are in the sales department; 39 people are in the marketing department; 32 people are in the finance department; 21 people are in the management department; 22 people are in the information technology department. In this valid questionnaire, there are a total of 190 male employees, accounting for 56.9% of the questionnaire survey; a total of 144 female employees, accounting for 43.1% of the questionnaire survey. There are 143 people with a college degree or below, accounting for 42.8%, 71 people with college degrees, accounting for 21.3%, and 120 people with college degrees or above, accounting for 35.9%. The average age is 30.90 years old. The average working week is 51.89 h.

### 3.2 Measuring tools

To ascertain the questionnaire’s reliability and validity, this investigation utilises established, mature scales. Prior to the survey, the English scale underwent a translation process that adhered to the standard procedure for translation and back-translation (60). The questionnaire-issuing team conducted multiple rounds of proofreading to ensure that the translated scale into Chinese was accurate. The entire study employed a 5-point Likert scale, with responses ranging from “strongly disagree” to “strongly agree” (as indicated on the questionnaire).

#### 3.2.1 Negative workplace gossip

Using the workplace gossip scale developed by Brady et al. (37), since this study only focuses on negative gossip, relevant items in this part are selected, a total of 10 items. Examples of questions include: “Have you talked about your boss’s conversations in the workplace. These conversations took place when your boss was not present or could not hear the conversation. When talking with colleagues, you have questioned your boss’s ability” and “Talk about a colleague’s conversation in a workplace context where your colleague is not physically present or able to hear the conversation, or criticize another colleague during a conversation.” The alpha reliability of the scale is 0.95.

#### 3.2.2 Professional commitment

The professional commitment scale developed by Suddaby et al. (61) was used, with a total of 7 items. Example questions include: “When I work, I identify with my job role” and “This work is now an important part of my career.” The alpha reliability of the scale is 0.93.

#### 3.2.3 Mindfulness

The mindfulness scale developed by Zheng et al. (62) was used, with a total of 18 items. Example questions such as: “When I am at work, my attention is entirely on work” and “I accept my unpleasant experiences at work.” The alpha reliability of the scale is 0.88.

#### 3.2.4 Superior trust

Using the scale developed by Lau et al. (63) according to the context of Chinese organizations, there are a total of 4 items. Example questions include: “Supervisors often assign important tasks to me” and “Supervisors were willing to rely on [their employees’] work-related judgments.” The alpha reliability of the scale is 0.85.

#### 3.2.5 Work engagement

The Work Engagement Scale developed by Rich et al. (16) includes three dimensions: emotion, cognition and physiology, with a total of 18 items. Example questions such as: “I work with intensity on my job” and “At work, my mind is focused on my job.” The alpha reliability of the scale is 0.92.

#### 3.2.6 Control variables

According to previous research, it has been found that the gender, age, and education level of employees will affect work engagement (64). At the same time, it was found that employees’ weekly working hours have different effects on work engagement (65, 66). Therefore, in order to verify the model more accurately, this study uses gender, age, education and weekly working hours as control variables.

### 3.3 Data analysis

This study used SPSS 25.0 for Harman’s one-way test, descriptive statistics, correlation analysis and multiple regression analysis, and Amos 22.0 was used for confirmatory factor analysis. When testing the mediating effect, this study uses the three-step method of Baron and Kenny (67) combined with the Bootstrap technique (using the PROCESS program-Model 4) (68) to estimate the confidence interval of the mediating effect. When testing moderated mediation, this study is based on the research of Edward and Lambert (69) and combines the Bootstrap technique (using the PROCESS program-Model 9) to test the value and significance of the difference between the indirect effect under high and low moderating variables.

## 4 Research results

### 4.1 Common method deviation test

In order to reduce the impact of common method bias, follow the multi-stage answering method suggested by Podsakoff et al. (70) to control the possible common method bias (70). At the data level of the survey results, a Harman single-factor test was performed on the collected data, and it was found that the variance explained by the first factor was 24.53%, which was less than the standard of 40% (70). From Table 1 that the fitting index of the confirmatory factor analysis of the single factor model did not pass the test (χ^2^ = 23015.23, df = 1,539, RMSEA = 0.20, SRMR = 0.29, CFI = 0.35, TLI = 0.33). Thus, the variables in this study do not exhibit any significant common method bias.

**Table 1:** Results of confirmatory factor analysis (*N* = 334).

Model	χ^2^	df	Δχ^2^	RMSEA	SRMR	CFI	TLI
Five-factor model (hypothesis)	6451.11	1,529		0.07	0.07	0.91	0.90
Four-factor model (A + B)	11727.33	1,533	5276.22***	0.12	0.12	0.78	0.72
Four-factor model (A + C)	11963.87	1,533	5512.76***	0.14	0.15	0.58	0.56
Four-factor model (A + D)	11253.23	1,533	4802.12***	0.13	0.13	0.61	0.59
Three-factor model (B + C + D)	12870.50	1,536	6419.39***	0.14	0.11	0.54	0.53
Two-factor model (A + B + C + D)	15870.13	1,538	9419.02***	0.17	0.18	0.43	0.41
Single-factor model (A + B + C + D + E)	23015.23	1,539	16564.12***	0.20	0.29	0.35	0.33

A: negative workplace gossip; B: professional commitment; C: mindfulness; D: superior trust; E: work engagement; and “+” indicates fusion.

### 4.2 Confirmatory factor analysis

The following fitting indices were chosen for examination in this study in order to assess the degree of model fitting. This study compares a number of competition models, and the results of the analysis are presented in Table 1. The model fit of the five-factor model in this study (χ2 = 6451.11, df = 1,529, RMSEA = 0.07, SRMR = 0.07, CFI = 0.91, TLI = 0.90) is better than other competing models. Furthermore, the test was passed by every fitness indicator comprising the five-factor model. This study concludes that all of the research variables are discernible on the basis of this.

### 4.3 Correlation analysis

There was a correlation between the variables and the control variables, as shown in Table 2. Table 2 shows that all variables are significantly linked, which gives us a starting point for testing the model’s hypothesis. A significant negative correlation between negative workplace gossip and work engagement (*r* = −0.26, *p* < 0.001), which supports investigating the negative impact of workplace gossip on work engagement.

**Table 2:** correlation coefficient of variables.

Variables	Mean	Standard deviation	1	2	3	4	5	6	7	8	9
1. Gender	0.43	0.50									
2. Age	30.90	6.74	0.10								
3. Educational level	11.86	2.74	0.07	0.27**							
4. Working hours weekly	51.89	11.95	−0.01	0.02	−0.08						
5. Negative workplace gossip	2.61	1.03	0.16**	0.10	−0.35***	−0.04	**(0.95)**				
6. Professional commitment	3.53	0.82	−0.01	−0.06	0.23***	0.04	−0.48***	**(0.93)**			
7. Mindfulness	3.64	1.09	−0.13*	−0.09	0.15**	0.09	−0.53***	0.35***	**(0.88)**		
8. Superior trust	4.49	0.74	0.04	0.03	0.06	0.19**	−0.14*	0.17***	0.27***	**(0.85)**	
9. Work engagement	3.97	0.93	−0.06	−0.10	0.06	−0.05	−0.26***	0.23***	0.54***	0.04	**(0.92)**

***p < 0.001, **p < 0.01, * *p* < 0.05. Bold values means the alpha reliability of variables.

### 4.4 Hypothesis testing results

#### 4.4.1 Test results of the main effect

As shown in Model 6 in Table 3, negative workplace gossip negatively impacts work engagement (β = −0.28, *p* < 0.001). Hypothesis 1 was supported.

**Table 3:** Hypothesis testing model.

Variables	Professional commitment					Work engagement			
	Model 1	Model 2	Model 3	Model 4	Model 5	Model6	Model 7	Model 8	Model 9
Gender	0.01	0.07	0.09	0.07	0.08	−0.01	−0.05	−0.02	0.02
Age	−0.00	−0.01	−0.01	0.00	0.00	−0.09	−0.08	−0.08	−0.05
Education	0.23***	0.07	0.05	0.03	0.03	−0.07	−0.03	−0.08	−0.04
Working hours weekly	0.06	0.03	0.02	0.02	0.01	−0.06	−0.06	−0.07	−0.07
Negative workplace gossip		−0.47***	−0.37***	−0.41***	−0.35***	−0.28***	.	−0.21**	−0.06*
Professional commitment					.		0.24***	0.15*	0.09*
Mindfulness			0.09		0.08				0.59*
Superior trust				0.09	0.06				0.19*
Negative workplace gossip* Mindfulness			0.14*		0.08*				0.03*
Negative workplace gossip* Superior trust				0.15**	0.13*				0.05*
*R^2^*	0.05	0.24	0.27	0.27	0.28	0.08	0.07	0.10	0.35
*F*	4.72**	20.74***	16.96***	17.15***	14.16***	5.86***	4.72***	5.93***	17.08***

***p < 0.001, **p < 0.01, *p < 0.05.

#### 4.4.2 Test results of the mediating effect

From Model 8 in Table 3, we can know that negative workplace gossip has a significant negative relationship with work engagement (β = −0.21, *p* < 0.01), and professional commitment has a significant positive relationship with work engagement (β = 0.15, *p* < 0.01), examined the indirect effect of negative workplace gossip on work engagement through professional commitment. In order to further clarify this indirect effect, Bootstrap (using the PROCESS program) (68) was used. Table 4 shows the mediating effect Bootstrap test. Direct and indirect effects of negative workplace gossip on work engagement are not zero at 95% confidence. Thus, professional commitment mediates the relationship between proactive negative workplace gossip and work engagement. Hypothesis 2 was supported.

**Table 4:** Bootstrap test of mediating role.

Mediating effect	Effect value	Standard error	95% confidence interval	
			Lower confidence limit	Upper confidence limit
Indirect effect	−0.06	0.03	−0.11	−0.01
Direct effect	−0.19	0.06	−0.30	−0.08

Bootstrap sample size *N* = 5,000.

#### 4.4.3 Test results of the moderating effect

**Figure 2:**
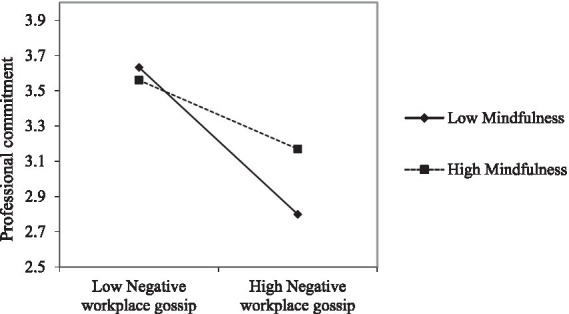
The moderating effect of mindfulness on the relationship between negative workplace gossip and professional commitment.

From Model 3 in Table 3, the interaction term between negative workplace gossip and mindfulness positively affects professional commitment (β = 0.14, *p* < 0.05). The Bootstrap test of the moderating effect is shown in Table 5. At the 95% confidence interval, when the level of mindfulness is low, the indirect effect of negative workplace gossip on professional commitment is higher (effect value is −0.40). When the level of mindfulness is high, the indirect effect of negative workplace gossip on professional commitment is lower (effect size is −0.19). In order to further clarify this moderating effect, the study was determined by using Aiken et al. (71) to adjust the high and low levels of the moderator. As shown in Figure 2, mindfulness reduces the negative relationship between workplace gossip and professional commitment. Hypothesis 3 is supported.

**Table 5:** Bootstrap test for the moderating effect of mindfulness.

Moderating effect	Effect value	Standard error	95% confidence interval	
			Lower confidence limit	Upper confidence limit
Low (−1SD)	−0.40	0.06	−0.52	−0.29
Medium	−0.29	0.05	−0.39	−0.20
High (+1SD)	−0.19	0.07	−0.33	−0.05

Bootstrap sample size *N* = 5,000.

**Figure 3:**
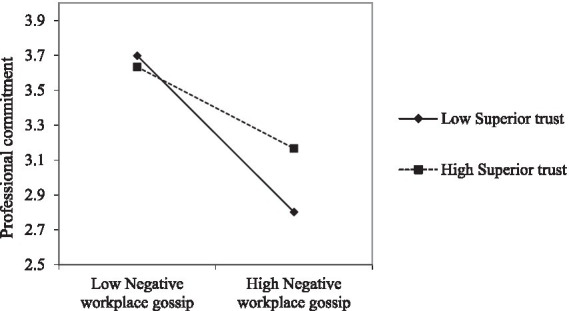
The moderating effect of superior trust on the relationship between negative workplace gossip and professional commitment.

Examining the moderating role of perceived superior trust. From Model 4 in Table 3, we can see that the interaction term between negative workplace gossip and perceived trust in superiors has a significant positive relationship with professional commitment (β = 0.15, *p* < 0.05). At the same time, the Bootstrap test of the moderating effect is shown in Table 6. At the 95% confidence interval, under a low level of perceived superior trust, the indirect effect of negative workplace gossip on professional commitment is higher (effect value is −0.43). Under high levels of perceived superior trust, the indirect effect of negative workplace gossip on professional commitment is low (effect size is −0.25). The study used Aiken et al. (71) to adjust the moderating variable’s high and low levels to clarify this effect. Figure 3 shows that negative workplace gossip and professional commitment are weaker when superiors are trusted. Hypothesis 4 was supported.

**Table 6:** Bootstrap test for the moderating effect of superior trust.

Moderating effect	Effect value	Standard error	95% confidence interval	
			Lower confidence limit	Upper confidence limit
Low (−1SD)	−0.43	0.05	−0.53	−0.34
Medium	−0.33	0.04	−0.41	−0.25
High (+1SD)	−0.25	0.05	−0.37	−0.15

Bootstrap sample size *N* = 5,000.

**Figure 4:**
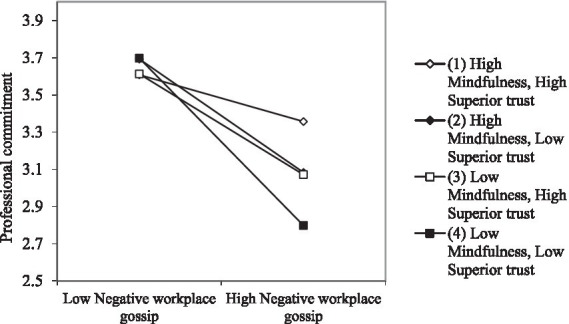
The dual moderating effect of mindfulness and superior trust on the relationship between negative workplace gossip and professional commitment.

From Model 5 in Table 3, we can see that the interaction term between negative workplace gossip and mindfulness has a significant positive relationship with professional commitment (β = 0.08, *p* < 0.05); at the same time, the interaction term between negative workplace gossip and perceived superior trust has a significant positive relationship with professional commitment (β = 0.13, *p* < 0.05). In this study, the Bootstrap test (68) was used to explore the dual moderation effect. As shown in Table 7, on the 95% confidence interval, when mindfulness and perceived superior trust are both low, the indirect effect of negative workplace gossip on professional commitment is relatively high (effect value −0.44); When perceived superior trust is high, or when mindfulness is high and perceived superior trust is low, the indirect effect of workplace negative gossip on professional commitment is reduced (the effect values are −0.29 and − 0.30 respectively); when mindfulness and perceived superior trust are both at a high level, the indirect effect of workplace negative gossip on professional commitment is low (effect value −0.14). It can be seen from Figure 4 that the higher the degree of mindfulness and perceived trust in superiors, the weaker the negative impact of negative workplace gossip on professional commitment.

**Table 7:** Bootstrap test for dual moderating effect between mindfulness and superior trust.

Moderating variable 1 (mindfulness)	Moderating variable 2 (superior trust)	Effect value	Standard error	95% confidence interval	
				Lower confidence limit	Upper confidence limit
Low (−1SD)	Low (−1SD)	−0.43	0.06	−0.55	−0.31
Low (−1SD)	High (+1SD)	−0.29	0.08	−0.44	−0.14
High (+1SD)	Low (−1SD)	−0.30	0.08	−0.45	−0.14
High (+1SD)	High (+1SD)	−0.14	0.07	−0.29	−0.01

Bootstrap sample size *N* = 5,000.

#### 4.4.4 Test results of the moderated mediating effect

To test whether mindfulness and perceived superior trust moderate the indirect effect of negative workplace gossip on work engagement via professional commitment. This study used the Bootstrap method to test the effect size of the indirect effect under high and low levels of moderating variables (69). As can be seen from Table 8, under high levels of mindfulness and high levels of perceived superior trust, the indirect effect of negative workplace gossip on work engagement through professional commitment is −0.02, with a value of [−0.07, −0.003] in the 95% confidence interval. Under low levels of mindfulness and low levels of perceived superior trust, the indirect effect of negative workplace gossip on work engagement through professional commitment is −0.07, with a 95% confidence interval of [−0.14, −0.02]. At the same time, when the level of mindfulness and perceived superior trust are inconsistent, the indirect effects of negative workplace gossip on work engagement through professional commitment are −0.05 and − 0.05, respectively, which are still significant in the 95% confidence interval. It can be seen that the higher the degree of mindfulness and perceived trust in superiors, the weaker negative workplace gossip through professional commitment to work engagement. Hypothesis 5 was supported.

**Table 8:** Bootstrap test with moderated mediating effect.

Independent variable	Moderator 1 (mindfulness)	Moderator 2 (superior trust)	Indirect effect	Standard error	95% confidence interval	
					Lower confidence limit	Upper confidence limit
Professional commitment	Low (−1SD)	Low (−1SD)	−0.07	0.03	−0.14	−0.02
	Low (−1SD)	High (+1SD)	−0.05	0.02	−0.09	−0.01
	High (+1SD)	Low (−1SD)	−0.05	0.02	−0.10	−0.01
	High (+1SD)	High (+1SD)	−0.02	0.02	−0.07	−0.003

Bootstrap sample size *N* = 5,000.

## 5 Discussion

### 5.1 Theoretical implications

Our findings contribute to the literature on negative workplace gossip, professional commitment, mindfulness, and superior trust in the following aspects:

First, utilizing the resource conservation theory, elucidates the adverse influence of negative workplace gossip on employees’ work engagement while enhancing comprehension of its impact. Negative workplace gossip is prevalent in organizational contexts, aligning with scholarly projections (25). Given the contemporary landscape in China and within organizational settings, employees now place greater emphasis on work quality, psychological fulfillment, and self-value realization. Previous studies focused on employees’ job performance, innovative behavior and creativity, etc. (7, 8, 10, 11). Our research found that workplace negative gossip will reduce employees’ commitment and engagement to work, which may better explain the importance of damaging workplace negative gossip. Consequently, the investigation of factors shaping work engagement holds considerable significance.

Second, through empirical analysis, this study confirmed the mediating function of professional commitment in the connection between negative workplace gossip and work engagement, thus enhancing our comprehension of the mechanisms behind the influence of workplace negativity on work engagement. Workplace negative gossip, viewed as a stressful situational occurrence, induces a perceived threat of resource loss for employees (18), subsequently impeding their professional commitment and work engagement. This further augments our understanding of the constructive role of professional commitment and introduces novel research variables for the examination of workplace negative gossip. The findings of this study also provide a new mechanism perspective for future research on workplace gossip.

Third, this study examines how external resources (trust in superiors) and internal resources (mindfulness) impact individuals and affirms that supervisor trust and employee mindfulness counteract the proliferation of negative workplace gossip’s adverse effects. This discovery answers the research call for investigating the multifaceted dynamics of workplace incivility and their influence on individuals (72, 73). Moreover, this study response their call and reveals that organizational trust appears to be a crucial boundary condition for workplace gossip (8). It broadens the parameters governing the influence of negative workplace gossip on employee work engagement.

### 5.2 Practical implications

This study centers on enhancing employees’ work engagement and strives to offer practical management recommendations for mitigating the adverse effects of workplace negative gossip. These suggestions are framed within the context of employees’ intrinsic and extrinsic resources.

#### 5.2.1 Establishing a positive organizational system and climate while mitigating negative gossip

Firstly, managers should be vigilant about the detrimental effects of negative workplace gossip and take proactive measures to suppress its impact on employees’ work engagement. Companies should promote a cultural and organizational environment characterized by fairness, justice, harmony, inclusivity, and mutual understanding (74). Additionally, they should bolster the development of systems that discourage the dissemination of negative gossip and penalize individuals spreading false information (10, 11). This not only fosters positive interpersonal relationships within the organization but also curtails the emergence of negative gossip at its roots.

#### 5.2.2 Boosting professional commitment for sustaining high work engagement

Furthermore, managers should proactively address the work-related effects of gossip and offer resources to employees through extensive communication (75), personalized support, team-building activities (76), and other means. This approach helps prevent employee burnout, which can impede their professional commitment and high work engagement levels. The findings are in line with previous research that has also called for communication to mitigate the damaging effects of workplace gossip (26).

#### 5.2.3 Trusting subordinates, improving employee mindfulness

Lastly, the mindfulness level of employees can influence their resilience against negative rumors. Therefore, the human resources department should consider mindfulness as an assessment criterion during recruitment, possibly by administering relevant psychological tests to job applicants (77). For example, Babalola et al. (14) found that employee mindfulness can regulate the impact of negative workplace gossip on customer service performance. Therefore, for employees with lower mindfulness, managers should implement supportive measures to help them navigate negative emotions in challenging work environments, effectively address incidents of negative gossip, and boost their work enthusiasm. Additionally, managers should prioritize building and nurturing trust-based relationships in daily management (78), offering positive psychological cues to employees to enhance their ability to handle workplace gossip.

## 6 Conclusion

As a key factor of employee performance and competitive advantage, work engagement has always been concerned by the field of workplace and organization management. This study, grounded in the context of pervasive negative workplace gossip and drawing upon the conservation of resources theory, delves into the internal mechanisms through which negative workplace gossip impacts work engagement, specifically through the lens of professional commitment. Furthermore, it aims to dissect the situational variables within this mechanism, taking into account the perspectives of both employees and their superiors, with a particular focus on mindfulness and the perception of trust in higher-ranking colleagues. From different perspectives, this paper proposes ways to alleviate the negative impact of negative workplace gossip, and expands new ideas in theory and new measures in practice to deal with negative workplace gossip.

## 7 Limitation and future research directions

This study is limited by subjective and objective conditions, and there are still the following shortcomings: First, the sample data was collected from front-line employees in mainland China, and the information was self-evaluated and reported by the employees themselves. This raises concerns about the universality and accuracy of the research results. Future studies could benefit from more diverse and objective data sources to enhance the generalizability of findings. Secondly, the research methodology employed in this study primarily consisted of questionnaire surveys, which can be considered a relatively singular research approach. It is suggested that future research could employ quasi-experimental methods to involve employees in relevant experiments, offering a more comprehensive exploration of negative workplace gossip and its impact on work engagement. Thirdly, this study reveals a dual-moderated mechanism based on individual psychological characteristics and perceived trust in superiors. Future research can consider organizational boundary conditions (such as organizational climate) and leadership styles [such as inclusive leadership (79)], so as to better provide practical suggestions for organizational management practices. Finally, future research may consider using different theories [such as cognitive-affective system theory (80)] to explore the impact of negative workplace gossip and work engagement.

## Data availability statement

The original contributions presented in the study are included in the article/supplementary material, further inquiries can be directed to the corresponding author.

## Author contributions

XC: Conceptualization, Data curation, Writing – review & editing. JD: Formal analysis, Methodology, Writing – review & editing. WW: Resources, Validation, Writing – original draft. LL: Conceptualization, Investigation, Writing – original draft, Writing – review & editing.
